# Annotations

**Published:** 1894-07-28

**Authors:** 


### ANNOTATIONS.

### Physiology and Collectivism.

The Rev. H. Jones-Henry, rector of Little Warley, in a paper read before the Essex Clerical Association, expresses the fear that collectivism, like the Philistines with Samson, is upon us, and that in no long time, by the active sympathy of the less intellectual clergy and the strenuous propaganda of collectivists whose hearts are stronger than their heads, society as a whole will become "collectivist" by compulsion. Human nature, however, we venture to think, will prove stronger than a too sympathetic clergy, and will resist even the onslaught of the well-meaning but watery-brained apostles of "collectivism." In our day physiology has taught us that she must be reckoned with by soft-hearted statesmen, and too eager reformers of their fellows. If there is one thing which physiology teaches more convincingly than another it is that the use of parts promotes their efficiency, and the disuse their atrophy. In other words, one of the most striking of physiological deductions is that a judicious strenuousness of every part of the human organism, brain included, produces the highest and most efficient form of man. Now collectivism, by making society responsible for the conduct of human affairs rather than individuals, aims a deadly blow at every form of strenuousness. It is impossible that any individual man shall do his utmost at anything, if he is convinced that society will do things better for him than he can do them for himself. Take away from all men the motive and the habit of working with their utmost energy, and you at once set up in them a process of brain atrophy. This is quite inevitable. Modern peoples may enter upon and may live through an era of collectivism; but if they do, they will certainly prove the sorriest generations of human beings which the world has ever yet seen. Physiology is the most absolute individualist. She insists upon every man's doing his "level best," his utmost, with every faculty he possesses. Failing to do his best, he is physiologically punished, and punished by the impairment and atrophy of the very faculties which he least uses.

### Mr. Gladstone's Sight.

Some public anxiety has not unnaturally been caused by the recent announcement that the pupil of Mr. Gladstone's eye, from which a cataract has been removed, is still obstructed by a film, and that a second operation will be necessary. Such an occurrence is by no means uncommon; and we are glad to be able to say that, as a rule, the second "operation" is of a trivial character, while the fact of its being required need not in the least interfere with the eventual excellence of the result. It often happens, when a cataract has been extracted, that the space formerly occupied by the opaque lens becomes the seat of a process of cell proliferation during the period of healing, or even of something which technically amounts to inflammation, and which, without being of sufficient severity to imperil vision, may leave a certain amount of deposit as it subsides. Such deposit usually shows itself as a "film" of greater or less density, occupying the pupillary area; and even a very slight film, apparently a mere cobweb, will materially diminish the acuteness of sight. These films were difficult and dangerous to deal with until the late Sir William Bowman, about 1853, introduced the method of tearing a central hole in them by the use of two needles at once, one acting against the other, in such a way as to avoid any dragging upon the region to which the films are attached. This kind of needling is, we presume, the "second operation" which is contemplated, and such a proceeding is scarcely attended by any appreciable degree of risk. It may be expected to restore perfect sight; and an eye on which it has been performed is left, in one important respect, in a better condition than one in which it has not been required. The needles establish a free communication between the aqueous and the vitreous humours; and this communication affords a great security against subsequent increase of tension.

### Barrack Children—An Object Lesson.

A responsible deputation waited upon Mr. Shaw-Lefevre last Monday to ask for a Royal Commission to inquire into the condition of those pauper children who are brought up by hundreds together in barracks, and to urge the desirability of the "cottage home" system. Almost all our leading philanthropists were present or expressed approval, including Sir John Gorst, the Bishop of Bedford, Canon Barnett, and many others; as also not a few men of science, who view the question at issue from the standpoint of physical science and economics. Among these latter were Sir Henry Roscoe, M.P., Dr. Wallace, of the Brentwood Schools, Mr. Henry C. Burdett, and Mr. Ernest Hart, editor of the *British Medical Journal*. Here, then, is a case in which sympathy and sense have met together, science and philanthropy have kissed each other. Those who have authority in this matter, and the reasoning public also, will do well to consider seriously the request (which was granted) for a Royal Commission and the grounds upon which it was based. The memorialists have issued an important memorandum of facts and arguments. These facts and arguments show, not only that the barrack system, the system of "collectivism," as it may be called, is a complete failure, but a dangerous and deadly failure. It is ruinous both to character and health. Why is it a failure? The memorialists tell us it is a failure because it does not develop physique or individual character in the children. The boys thus brought up are cowards and backboneless; the girls are dull and ignorant, and fall a prey to the seducer by scores as soon as they are outside the schools and set to fight their own battles in the world. All this is because they live in masses, and all individualism of character is crushed out of them. The cottage home system is undoubtedly the one hope of fatherless and motherless pauper children. Enforced "collectivism" is their mental and moral destruction. Brought up in barracks as they are, they start the race of separate life without a chance. It is not fair; it is not English not to give them a chance. No doubt many arguments such as "costliness" and so forth will be urged on the other side. But this argument, that the barrack-bred child is never given anything like a fair chance to make his way in the world should outweigh every other consideration. He must be given a chance. No Government at the present day should dare to deny him one.

